# Supplementary material for: Tests for segregation distortion in higher ploidy F1 populations
Source: G3 (Bethesda). 2025 Sep 15;15(11):jkaf212. doi: 10.1093/g3journal/jkaf212 (PMC12611257; doi:10.1093/g3journal/jkaf212)
Supplement: jkaf212_Supplementary_Data [file jkaf212_supplementary_data.pdf]

Supplement to: "Tests for segregation distortion in higher ploidy F1 populations"

David Gerard[1*], Guilherme Bovi Ambrosano[2],
Guilherme da Silva Pereira[3], and Antonio Augusto Franco Garcia[2]

[1]Department of Mathematics and Statistics, American University, Washington, DC, 20016-8002, USA

[2]Department of Genetics, Luiz de Queiroz College of Agriculture, University of São Paulo, Piracicaba, 13418-900, Brazil

[3]Department of Agronomy, Federal University of Viçosa, Viçosa, Minas Gerais, 36570-900, Brazil

[*]Corresponding author: dgerard@american.edu

Abstract

This document contains additional theoretical considerations, derivations, and figures to supplement the manuscript "Tests for segregation distortion in higher ploidy F1 populations".

S1 Proof of Theorem 1

Proof. For p_0, the minor allele is not in the gamete if it is not one of the i double reduced alleles (probability $\binom{K-1}{i}/\binom{K}{i}$) and it is not one of the non-double-reduced alleles (probability $\binom{K-1-i}{K/2-2i}/\binom{K-i}{K/2-2i}$). We marginalize over the number of double reduced pairs to obtain

$$p_0 = \sum_{i=0}^{\lfloor K/4 \rfloor} \alpha_i \frac{\binom{K-1}{i}\binom{K-1-i}{K/2-2i}}{\binom{K}{i}\binom{K-i}{K/2-2i}} \tag{S1}$$

$$= \sum_{i=0}^{\lfloor K/4 \rfloor} \alpha_i \frac{(K-1)!}{i!(K-1-i)!} \frac{(K-1-i)!}{(K/2-2i)!(K/2+i-1)!} \frac{i!(K-i)!}{K!} \frac{(K/2-2i)!(K/2+i)!}{(K-i)!} \tag{S2}$$

$$= \sum_{i=0}^{\lfloor K/4 \rfloor} \alpha_i \frac{K/2+i}{K} \tag{S3}$$

$$= \frac{1}{2}\alpha_0 + \sum_{i=1}^{\lfloor K/4 \rfloor} \alpha_i \frac{K/2+i}{K} \tag{S4}$$

$$= \frac{1}{2}\left(1 - \sum_{i=1}^{\lfloor K/4 \rfloor} \alpha_i\right) + \sum_{i=1}^{\lfloor K/4 \rfloor} \alpha_i \frac{K/2+i}{K} \tag{S5}$$

$$= \frac{1}{2} + \sum_{i=1}^{\lfloor K/4 \rfloor} \alpha_i \left(\frac{K/2+i}{K} - \frac{1}{2}\right) \tag{S6}$$

$$= \frac{1}{2} + \sum_{i=1}^{\lfloor K/4 \rfloor} \alpha_i \frac{i}{K}. \tag{S7}$$

Similarly, for p_1, the minor allele shows up once in the gamete if it is not one of the i double reduced alleles (probability $\binom{K-1}{i}/\binom{K}{i}$) and it *is* one of the non-double-reduced alleles (probability $\binom{K-1-i}{K/2-2i-1}/\binom{K-i}{K/2-2i}$). We marginalize over the number of double reduced pairs to obtain

$$
\begin{aligned}
p_1 &= \sum_{i=0}^{\lfloor K/4 \rfloor} \alpha_i \frac{\binom{K-1}{i}\binom{K-1-i}{K/2-2i-1}}{\binom{K}{i}\binom{K-i}{K/2-2i}} && \text{(S8)}\\
&= \sum_{i=0}^{\lfloor K/4 \rfloor} \alpha_i \frac{(K-1)!}{i!(K-1-i)!} \frac{(K-1-i)!}{(K/2-2i-1)!(K/2+i)!} \frac{i!(K-i)!}{K!} \frac{(K/2-2i)!(K/2+i)!}{(K-i)!} && \text{(S9)}\\
&= \sum_{i=0}^{\lfloor K/4 \rfloor} \alpha_i \frac{K/2-2i}{K} && \text{(S10)}\\
&= \frac{1}{2}\alpha_0 + \sum_{i=1}^{\lfloor K/4 \rfloor} \alpha_i \frac{K/2-2i}{K} && \text{(S11)}\\
&= \frac{1}{2}\left(1 - \sum_{i=1}^{\lfloor K/4 \rfloor} \alpha_i\right) + \sum_{i=1}^{\lfloor K/4 \rfloor} \alpha_i \frac{K/2-2i}{K} && \text{(S12)}\\
&= \frac{1}{2} + \sum_{i=1}^{\lfloor K/4 \rfloor} \alpha_i \left(\frac{K/2-2i}{K} - \frac{1}{2}\right) && \text{(S13)}\\
&= \frac{1}{2} - 2\sum_{i=1}^{\lfloor K/4 \rfloor} \alpha_i \frac{i}{K} && \text{(S14)}
\end{aligned}
$$

For p_2, we could derive its value from $p_0 + p_1 + p_2 = 1$. Or we can derive it from first principles. The minor allele shows up twice in the gamete if it is one of the i double reduced alleles (probability $\binom{K-1}{i-1}/\binom{K}{i}$). Marginalizing over the number of double reduced pairs, we have

$$
\begin{aligned}
p_2 &= \sum_{i=1}^{\lfloor K/4 \rfloor} \alpha_i \frac{\binom{K-1}{i-1}}{\binom{K}{i}} && \text{(S15)}\\
&= \sum_{i=1}^{\lfloor K/4 \rfloor} \alpha_i \frac{(K-1)!}{(i-1)!(K-i)!} \frac{i!(K-i)!}{K!} && \text{(S16)}\\
&= \sum_{i=1}^{\lfloor K/4 \rfloor} \alpha_i \frac{i}{K}. && \text{(S17)}
\end{aligned}
$$

□

S2 Our model for the gamete frequencies for ploidies 2, 4, 6, 8, 10, and 12

Given a parent dosage, ℓ, below we list the model for the gamete frequencies $\boldsymbol{p} = (p_0, \ldots, p_{K/2})$ at a given ploidy K. Note that $\sum \gamma_i = 1$.

Ploidy = 2

- $\ell = 0$: $(1, 0)$
- $\ell = 1$: $(1, 1)/2$
- $\ell = 2$: $(0, 1)$

Ploidy = 4

- $\ell = 0$: $(1, 0, 0)$
- $\ell = 1$: $(\frac{1}{2} + \beta, \frac{1}{2} - 2\beta, \beta)$
- $\ell = 2$: $\gamma_1 \frac{1}{4}(1, 2, 1) + \gamma_2(0, 1, 0)$
- $\ell = 3$: $(\beta, \frac{1}{2} - 2\beta, \frac{1}{2} + \beta)$
- $\ell = 4$: $(0, 0, 1)$

Ploidy = 6

- $\ell = 0$: $(1, 0, 0, 0)$
- $\ell = 1$: $(\frac{1}{2} + \beta, \frac{1}{2} - 2\beta, \beta, 0)$
- $\ell = 2$: $\gamma_1 \frac{1}{4}(1, 2, 1, 0) + \gamma_2(0, 1, 0, 0)$
- $\ell = 3$: $\gamma_1 \frac{1}{8}(1, 3, 3, 1) + \gamma_2 \frac{1}{2}(0, 1, 1, 0)$
- $\ell = 4$: $\gamma_1 \frac{1}{4}(0, 1, 2, 1) + \gamma_2(0, 0, 1, 0)$
- $\ell = 5$: $(0, \beta, \frac{1}{2} - 2\beta, \frac{1}{2} + \beta)$
- $\ell = 6$: $(0, 0, 0, 1)$

Ploidy = 8

- $\ell = 0$: $(1, 0, 0, 0, 0)$
- $\ell = 1$: $(\frac{1}{2} + \beta, \frac{1}{2} - 2\beta, \beta, 0, 0)$
- $\ell = 2$: $\gamma_1 \frac{1}{4}(1, 2, 1, 0, 0) + \gamma_2(0, 1, 0, 0, 0)$
- $\ell = 3$: $\gamma_1 \frac{1}{8}(1, 3, 3, 1, 0) + \gamma_2 \frac{1}{2}(0, 1, 1, 0, 0)$
- $\ell = 4$: $\gamma_1 \frac{1}{16}(1, 4, 6, 4, 1) + \gamma_2 \frac{1}{4}(0, 1, 2, 1, 0) + \gamma_3(0, 0, 1, 0, 0)$
- $\ell = 5$: $\gamma_1 \frac{1}{8}(0, 1, 3, 3, 1) + \gamma_2 \frac{1}{2}(0, 0, 1, 1, 0)$
- $\ell = 6$: $\gamma_1 \frac{1}{4}(0, 0, 1, 2, 1) + \gamma_2(0, 0, 0, 1, 0)$
- $\ell = 7$: $(0, 0, \beta, \frac{1}{2} - 2\beta, \frac{1}{2} + \beta)$
- $\ell = 8$: $(0, 0, 0, 0, 1)$

Ploidy = 10

- $\ell = 0$: $(1, 0, 0, 0, 0, 0)$
- $\ell = 1$: $(\frac{1}{2} + \beta, \frac{1}{2} - 2\beta, \beta, 0, 0, 0)$
- $\ell = 2$: $\gamma_1 \frac{1}{4}(1, 2, 1, 0, 0, 0) + \gamma_2(0, 1, 0, 0, 0, 0)$

- $\ell = 3$: $\gamma_1 \frac{1}{8}(1,3,3,1,0,0) + \gamma_2 \frac{1}{2}(0,1,1,0,0,0)$
- $\ell = 4$: $\gamma_1 \frac{1}{16}(1,4,6,4,1,0) + \gamma_2 \frac{1}{4}(0,1,2,1,0,0) + \gamma_3(0,0,1,0,0,0)$
- $\ell = 5$: $\gamma_1 \frac{1}{32}(1,5,10,10,5,1) + \gamma_2 \frac{1}{8}(0,1,3,3,1,0) + \gamma_3 \frac{1}{2}(0,0,1,1,0,0)$
- $\ell = 6$: $\gamma_1 \frac{1}{16}(0,1,4,6,4,1) + \gamma_2 \frac{1}{4}(0,0,1,2,1,0) + \gamma_3(0,0,0,1,0,0)$
- $\ell = 7$: $\gamma_1 \frac{1}{8}(0,0,1,3,3,1) + \gamma_2 \frac{1}{2}(0,0,0,1,1,0)$
- $\ell = 8$: $\gamma_1 \frac{1}{4}(0,0,0,1,2,1) + \gamma_2(0,0,0,0,1,0)$
- $\ell = 9$: $(0,0,0,\beta,\frac{1}{2}-2\beta,\frac{1}{2}+\beta)$
- $\ell = 10$: $(0,0,0,0,0,1)$

Ploidy = 12

- $\ell = 0$: $(1,0,0,0,0,0,0)$
- $\ell = 1$: $(\frac{1}{2}+\beta,\frac{1}{2}-2\beta,\beta,\beta,0,0,0,0)$
- $\ell = 2$: $\gamma_1 \frac{1}{4}(1,2,1,0,0,0,0) + \gamma_2(0,1,0,0,0,0,0)$
- $\ell = 3$: $\gamma_1 \frac{1}{8}(1,3,3,1,0,0,0) + \gamma_2 \frac{1}{2}(0,1,1,0,0,0,0)$
- $\ell = 4$: $\gamma_1 \frac{1}{16}(1,4,6,4,1,0,0) + \gamma_2 \frac{1}{4}(0,1,2,1,0,0,0) + \gamma_3(0,0,1,0,0,0,0)$
- $\ell = 5$: $\gamma_1 \frac{1}{32}(1,5,10,10,5,1,0) + \gamma_2 \frac{1}{8}(0,1,3,3,1,0,0) + \gamma_3 \frac{1}{2}(0,0,1,1,0,0,0)$
- $\ell = 6$: $\gamma_1 \frac{1}{64}(1,6,15,20,15,6,1)+\gamma_2 \frac{1}{16}(0,1,4,6,4,1,0)+\gamma_3 \frac{1}{4}(0,0,1,2,1,0,0)+\gamma_4(0,0,0,1,0,0,0)$
- $\ell = 7$: $\gamma_1 \frac{1}{32}(0,1,5,10,10,5,1) + \gamma_2 \frac{1}{8}(0,0,1,3,3,1,0) + \gamma_3 \frac{1}{2}(0,0,0,1,1,0,0)$
- $\ell = 8$: $\gamma_1 \frac{1}{16}(0,0,1,4,6,4,1) + \gamma_2 \frac{1}{4}(0,0,0,1,2,1,0) + \gamma_3(0,0,0,0,1,0,0)$
- $\ell = 9$: $\gamma_1 \frac{1}{8}(0,0,0,1,3,3,1) + \gamma_2 \frac{1}{2}(0,0,0,0,1,1,0)$
- $\ell = 10$: $\gamma_1 \frac{1}{4}(0,0,0,0,1,2,1) + \gamma_2(0,0,0,0,0,1,0)$
- $\ell = 11$: $(0,0,0,0,\beta,\frac{1}{2}-2\beta,\frac{1}{2}+\beta)$
- $\ell = 12$: $(0,0,0,0,0,0,1)$

S3 Supplementary tables and figures

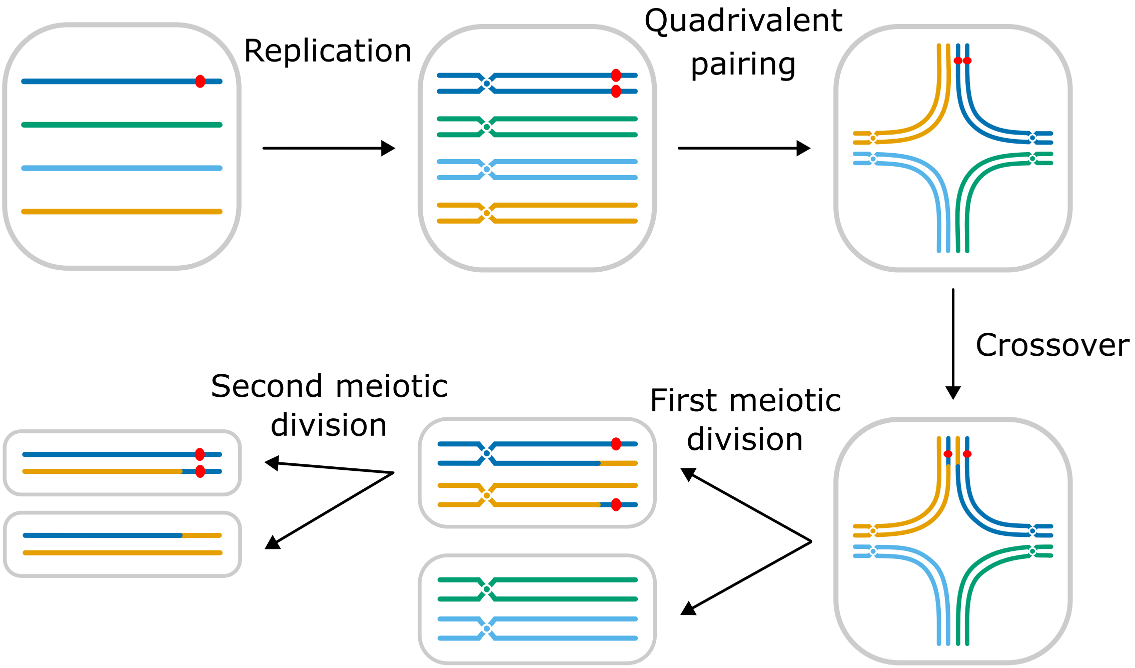

Figure S1: A graphical illustration of double reduction at a simplex locus in a tetraploid parent. The locus of interest carries a red allele on the dark blue chromosome. After replication, the chromosomes pair in a quadrivalent formation, with the SNP of interest located in the dark blue/orange and light blue/green pairings. Chromosome-colored dots represent the centromeres. A crossover occurs, resulting in the exchange of segments between the orange and blue chromosomes, including the allele of interest. During the first and second meiotic divisions, the sister chromatid segments containing the red allele may migrate to the same gamete, demonstrating that a simplex parent can produce duplex gametes.

Parent Dosage	Ploidy					
	2	4	6	8	10	12
0	(1,0)	(1,0,0)	(1,0,0,0)	(1,0,0,0,0)	(1,0,0,0,0,0)	(1,0,0,0,0,0,0)
1	(1,1)/2	(1,1,0)/2	(1,1,0,0)/2	(1,1,0,0,0)/2	(1,1,0,0,0,0)/2	(1,1,0,0,0,0,0)/2
2	(0,1)	(1,2,1)/4<br>(0,1,0)	(1,2,1,0)/4<br>(0,1,0,0)	(1,2,1,0,0)/4<br>(0,1,0,0,0)	(1,2,1,0,0,0)/4<br>(0,1,0,0,0,0)	(1,2,1,0,0,0,0)/4<br>(0,1,0,0,0,0,0)
3	-	(0,1,1)/2	(1,3,3,1)/8<br>(0,1,1,0)/2	(1,3,3,1,0)/8<br>(0,1,1,0,0)/2	(1,3,3,1,0,0)/8<br>(0,1,1,0,0,0)/2	(1,3,3,1,0,0,0)/8<br>(0,1,1,0,0,0,0)/2
4	-	(0,0,1)	(0,1,2,1)/4<br>(0,0,1,0)	(1,4,6,4,1)/16<br>(0,1,2,1,0)/4<br>(0,0,1,0,0)	(1,4,6,4,1,0)/16<br>(0,1,2,1,0,0)/4<br>(0,0,1,0,0,0)	(1,4,6,4,1,0,0)/16<br>(0,1,2,1,0,0,0)/4<br>(0,0,1,0,0,0,0)
5	-	-	(0,0,1,1)/2	(0,1,3,3,1)/8<br>(0,0,1,1,0)/2	(1,5,10,10,5,1)/32<br>(0,1,3,3,1,0)/8<br>(0,0,1,1,0,0)/2	(1,5,10,10,5,1,0)/32<br>(0,1,3,3,1,0,0)/8<br>(0,0,1,1,0,0,0)/2
6	-	-	(0,0,0,1)	(0,0,1,2,1)/4<br>(0,0,0,1,0)	(0,1,4,6,4,1)/16<br>(0,0,1,2,1,0)/4<br>(0,0,0,1,0,0)	(1,6,15,20,15,6,1)/64<br>(0,1,4,6,4,1,0)/16<br>(0,0,1,2,1,0,0)/4<br>(0,0,0,1,0,0,0)
7	-	-	-	(0,0,0,1,1)/2	(0,0,1,3,3,1)/8<br>(0,0,0,1,1,0)/2	(0,1,5,10,10,5,1)/32<br>(0,0,1,3,3,1,0)/8<br>(0,0,0,1,1,0,0)/2
8	-	-	-	(0,0,0,0,1)	(0,0,0,1,2,1)/4<br>(0,0,0,0,1,0)	(0,0,1,4,6,4,1)/16<br>(0,0,0,1,2,1,0)/4<br>(0,0,0,0,1,0,0)
9	-	-	-	-	(0,0,0,0,1,1)/2	(0,0,0,1,3,3,1)/8<br>(0,0,0,0,1,1,0)/2
10	-	-	-	-	(0,0,0,0,0,1)	(0,0,0,0,1,2,1)/4<br>(0,0,0,0,0,1,0)
11	-	-	-	-	-	(0,0,0,0,0,1,1)/2
12	-	-	-	-	-	(0,0,0,0,0,0,1)

Table S1: Gamete frequencies for true allopolyploids. Each cell contains the possible gamete frequencies $(p_0, p_1, \ldots, p_{K/2})$ of a true allopolyploid for a given ploidy K (column) and a given parental dosage (row).

Meiosis Model	Ploidy				
	4	6	8	10	12
PRCS	$1/28 \approx 0.03571$	$1/22 \approx 0.04545$	$1/20 = 0.05$	$1/19 \approx 0.05263$	$5/92 \approx 0.05435$
CES	$1/24 \approx 0.04167$	$1/20 = 0.05$	$3/56 \approx 0.05357$	$1/18 \approx 0.05556$	$5/88 \approx 0.05682$

Table S2: Upper bounds on β from (7) under either the complete equational segregation model (CES) (where whole arms of sister chromatids are exchanged) [Mather, 1935] or the pure random chromatid segregation model (PRCS) (where chromatids act independently and segregate into gametes with equal probability) [Haldane, 1930].

n	Ploidy	Read Depth	ℓ_1	ℓ_2	Type I Error Rate
200	8	Inf	0	2	0.38
200	8	Inf	8	2	0.35
200	8	Inf	0	6	0.34
200	8	Inf	8	6	0.28
200	6	Inf	0	2	0.26
200	6	Inf	0	4	0.26
200	6	Inf	6	2	0.26
200	6	Inf	6	4	0.20

Table S3: Eight simulation scenarios for `segtest` with poor type I error control under moderate double reduction (Section Robustness to double reduction). All scenarios involve duplex-by-nullplex crosses, known genotypes, and large sample sizes.

SNP	`mappoly`	`segtest`	`mappoly` All	`segtest` Less	`segtest` Null Alleles
S8_2301244	0.01	0.21	0.19		
S8_2301256	0.0034	0.11	0.12		
S8_5495374	0.0053	0.9	0.18		
S8_16038595	0.031	0.33	0.3		
S8_460771	0.12	8.9E-08		0.19	0.053
S8_2212186	0.13	2.8E-10		0.18	0.00012
S8_4866982	0.51	1.7E-07		0.22	1
S8_18370562	0.36	0.045		0.3	0.62

Table S4: P-values for SNPs where the results from `mappoly` and `segtest` differ. The "`mappoly`" and "`segtest`" columns show p-values under each method's default behavior. The "`mappoly` All" column shows p-values from `mappoly` when including individuals that are normally filtered out based on maximum posterior probability. The "`segtest` Less" column shows p-values from `segtest` after removing individuals with low reference read counts. The "`segtest` Null Alleles" columns shows the p-values from `segtest` when using an outlier distribution that accounts for null alleles.

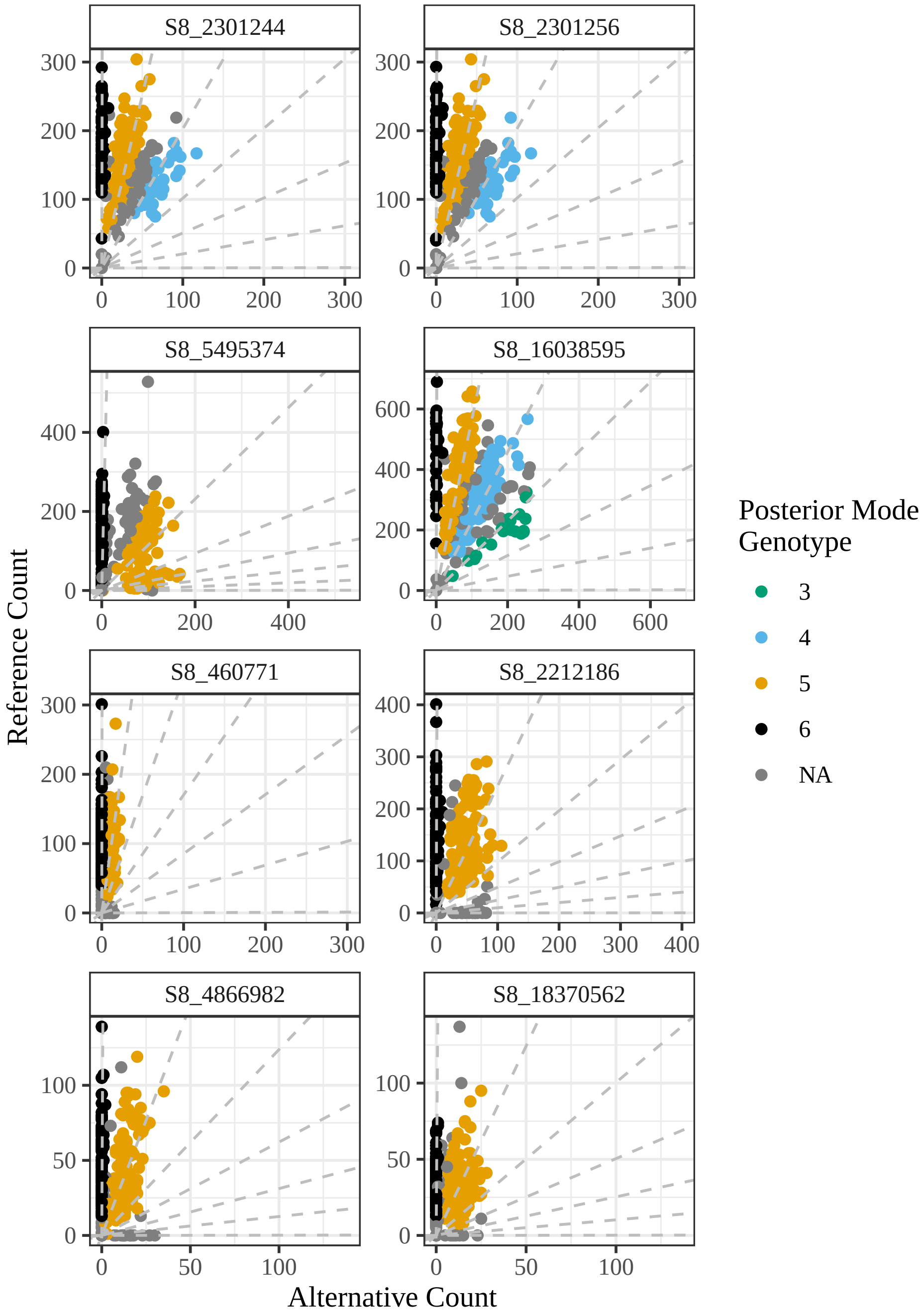

Figure S2: Genotype plots [Gerard et al., 2018] for a sample of SNPs where the results from `mappoly` and `segtest` differ. Alternative allele counts are on the x-axis, and reference allele counts are on the y-axis. Individuals are color-coded by posterior mode genotype. Missing values ("NA") indicate individuals that were filtered out by `mappoly`, though `segtest` includes all individuals in its test. The top four SNPs have low p-values from `mappoly` and high p-values from `segtest`. The bottom four SNPs show the opposite pattern, with low p-values from `segtest` and high p-values from `mappoly`.

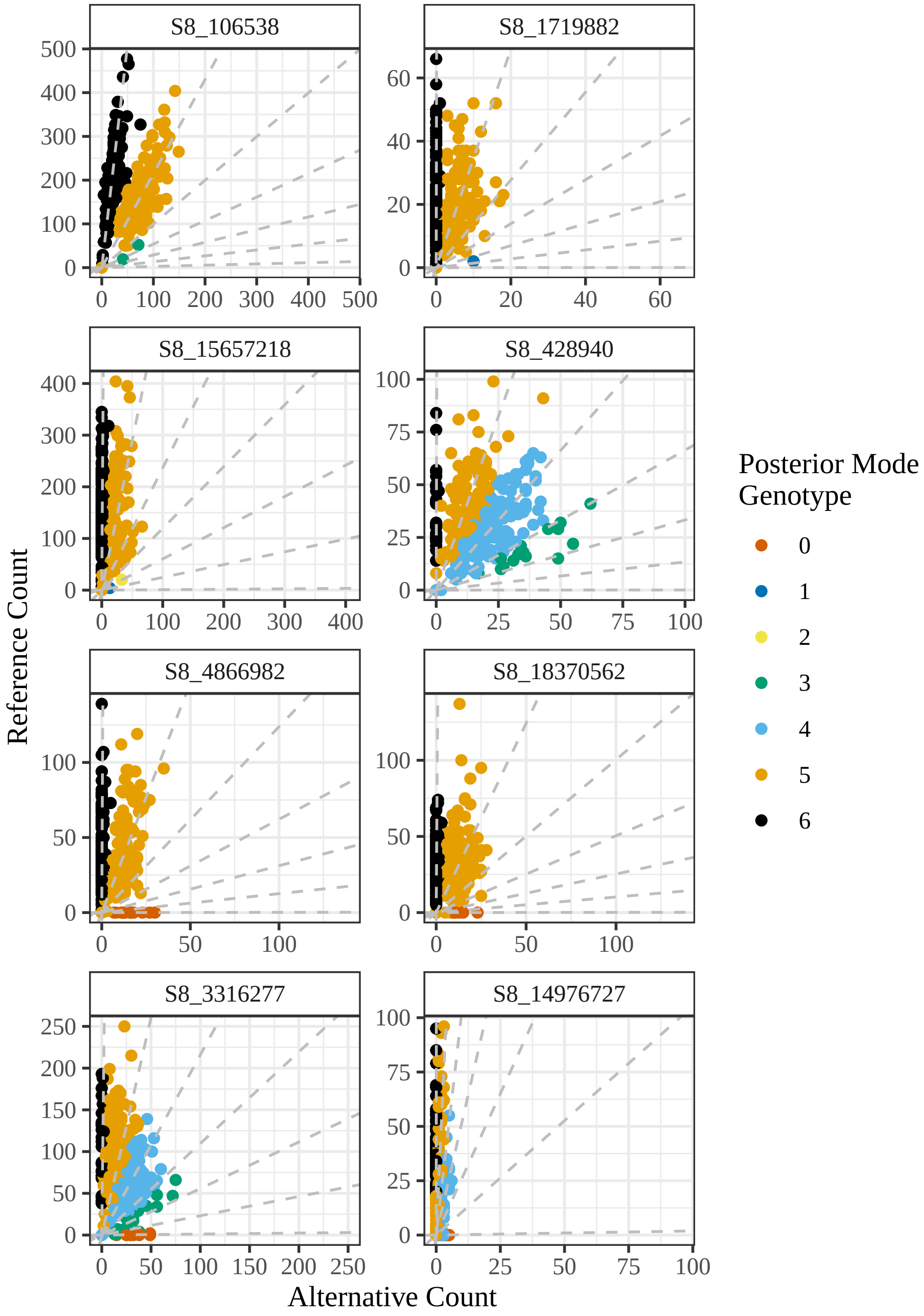

Figure S3: Genotype plots [Gerard et al., 2018] for a sample of SNPs where the results from `polymapR` and `segtest` differ. Alternative allele counts are on the x-axis, and reference allele counts are on the y-axis. Individuals are color-coded by posterior mode genotype. The top four SNPs have low p-values from `polymapR` and high p-values from `segtest`. The bottom four SNPs show the opposite pattern, with low p-values from `segtest` and high p-values from `polymapR`.

SNP	polymapR	segtest	segtest Less	segtest Null Alleles
S8_106538	0.0047	0.2		
S8_1719882	0.0096	0.56		
S8_15657218	0.0073	0.55		
S8_428940	0.0012	0.8		
S8_4866982	0.41	1.7E-07	0.19	1
S8_18370562	0.63	0.045	0.57	0.62
S8_3316277	1	0.00046	0.35	0.37
S8_14976727	0.93	1.4E-10	0.036	0.03

Table S5: P-values for SNPs where the results from `polymapR` and `segtest` differ. The "`polymapR`" and "`segtest`" columns show p-values under each method's default behavior. The "`segtest` Less" column shows p-values from `segtest` after removing individuals with low reference read counts. The "`segtest` Null Alleles" columns shows the p-values from `segtest` when using an outlier distribution that accounts for null alleles.

		Genotype						
SNP	Scenario	0	1	2	3	4	5	6
S8_106538	`polymapR` Alt	0	0	0	0	0	.420	.580
	`polymapR` Null	0	0	0	0	0	.500	.500
	`segtest` Alt	0	0	0	.002	.023	.396	.578
	`segtest` Null	0	0	0	0	.037	.426	.537
S8_15657218	`polymapR` Alt	0	0	0	0	0	.425	.575
	`polymapR` Null	0	0	0	0	0	.500	.500
	`segtest` Alt	0	0	.006	0	.036	.376	.582
	`segtest` Null	.001	.001	.001	.001	.051	.398	.546
S8_1719882	`polymapR` Alt	0	0	0	0	0	.427	.573
	`polymapR` Null	0	0	0	0	0	.500	.500
	`segtest` Alt	0	0	.003	0	.024	.393	.580
	`segtest` Null	0	0	0	0	.047	.407	.545
S8_428940	`polymapR` Alt	0	0	0	.064	.447	.394	.095
	`polymapR` Null	0	0	0	.050	.450	.450	.050
	`segtest` Alt	0	0	.003	.072	.446	.382	.097
	`segtest` Null	0	0	0	.091	.409	.409	.091

Table S6: Estimated genotype frequencies under different methods and hypotheses for four SNPs where `segtest` indicates no segregation distortion, but `polymapR` indicates strong segregation distortion. "`polymapR` Alt" contains `polymapR`'s estimated empirical genotype frequencies from genotype posterior probabilities [see Gerard et al., 2025, for a summary]. "`polymapR` Null" contains the expected genotype frequencies under polysomic inheritance with bivalent pairing. "`segtest` Alt" contains the genotype frequencies estimated using the method of Li [2011]. And "`segtest` Null" contains the estimated genotype frequencies under the null model from Section Models for genotype frequencies.

References

D. Gerard, L. F. V. Ferrão, A. A. F. Garcia, and M. Stephens. Genotyping polyploids from messy sequencing data. *Genetics*, 210(3):789–807, 2018. doi: 10.1534/genetics.118.301468.

D. Gerard, M. Thakkar, and L. F. V. Ferrão. Tests for segregation distortion in tetraploid F1 populations. *Theoretical and Applied Genetics*, 138(30), 2025. doi: 10.1007/s00122-025-04816-z.

J. Haldane. Theoretical genetics of autopolyploids. *Journal of Genetics*, 22(3):359–372, 1930. doi: 10.1007/BF02984197.

H. Li. A statistical framework for SNP calling, mutation discovery, association mapping and population genetical parameter estimation from sequencing data. *Bioinformatics*, 27(21):2987, 2011. doi: 10.1093/bioinformatics/btr509.

K. Mather. Reductional and equational separation of the chromosomes in bivalents and multivalents. *Journal of Genetics*, 30(1):53–78, 1935. doi: 10.1007/BF02982205.
